# PNA5, a glycosylated angiotensin-(1−7) mas receptor agonist for vascular dementia: A two species toxicology and toxicokinetic study

**DOI:** 10.1016/j.toxrep.2025.102151

**Published:** 2025-10-28

**Authors:** Christina Hoyer-Kimura, John P. Konhilas, Meredith Hay

**Affiliations:** a University of Arizona College of Medicine, Tucson, AZ, USA; b Sarver Heart Center, Tucson, AZ, USA; c Evelyn F. McKnight Brain Institute, Tucson, AZ, USA; d ProNeurogen, Inc, Tucson, AZ, USA

**Keywords:** Glycosylated-Angiotensin-(1−7) (Ang-1–7), Drug development, Vascular contributions to cognitive impairment and dementia

## Abstract

PNA5 is a novel pleotropic anti-inflammatory glycosylated-Angiotensin-(1−7) peptide derivative with outstanding brain penetration and enhanced bioavailability. PNA5, via the Mas receptor, decreases brain and cerebrovascular inflammation, reduces reactive oxygen species and inflammatory cytokines production, improves cerebral blood flow, and restores cognitive function in our mouse model of VCID (Vascular contributions to cognitive impairment and dementia). This study evaluated the potential systemic and local toxicity and toxicokinetic (TK) of PNA5 following daily subcutaneous administration in Sprague Dawley rats and Beagle dogs for 28 consecutive days. PNA5 was given at 1, 5, 40 mg/kg/day to rats (n = 12) and at 1, 5, and 20 mg/kg/day in dogs (n = 6) once daily for 28 consecutive days. Blood samples were collected on days 1 and 28 for TK analysis. PNA5 was well tolerated at all doses in both species, with no test article-related mortality or adverse effects. Systemic exposure to PNA5 appeared to be independent of sex. In rats, Cmax and AUC_0–2hr_ values increased with increasing doses. Systemic exposure to PNA5 in rats was greater on day 28 compared to day 1 following repeated administration of PNA5. In dogs, Cmax values increased less than dose-proportionally, and AUC_0–2hr_ increased approximately dose-proportionally on days 1 and 28. Systemic exposure to PNA5 in dogs was similar on days 1 and 28 following repeated administration. These results show that PNA5 has no toxicological effects at the highest doses tested in rats or dogs and is well tolerated with repeated exposure for 28 days. Accumulation was observed in rats but not in dogs.

## Highlight

• PNA5: a novel pleotropic glycosylated-Angiotensin-(1−7) peptide derivative.
• PNA5 was well tolerated at all doses in rats and dogs, with no adverse effects.
• C_max_ and AUC_0–2hr_ are dose-proportional in rats.
• C_max_ is dose proportional, and AUC_0–2hr_ is dose-proportional in dogs.
• Accumulation was observed in rats but not in dogs.

## Graphical Abstract

**Figure:**
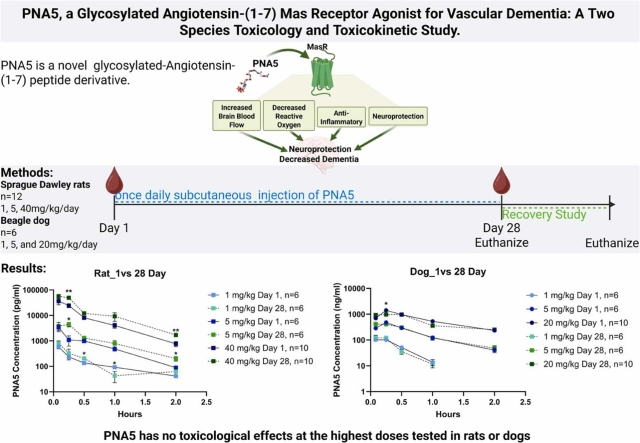

## 1 Introduction

The peptide PNA5 is a novel glycosylated Angiotensin-(1−7) (Ang-(1−7)) Mas receptor agonist with enhanced pharmacokinetic and pharmacodynamic (PK/PD) properties. This agonist was designed to improve bioavailability, stability, and brain penetration of Ang-(1−7) [1], [2]. Glycosylation of Angiotensin 1–7 (DRVYIHP) replaces the seventh residue (proline) with a serine that has a glucose sugar moiety attached and is amidated on the C-terminus, resulting in Ang-1–6-Ser-O-Glu-NH_2_ (PNA5). The molecular weight of PNA5 is 1278 g/mol.

PNA5 has outstanding brain penetration, enhanced bioavailability, decreases brain and cerebrovascular inflammation, improves cerebral blood flow and restores cognitive function in our preclinical model of VCID (Vascular contributions to cognitive impairment and dementia) [1], [2], [3], [4], [5], [6], [7]. Within the brain, the Mas receptor (MasR) is expressed on neurons, glia, and vascular endothelial cells [8], [9], [10]. Activation of Mas decreases reactive oxygen species (ROS)and brain inflammation, increases cerebral circulation [11], [12], [13] increases the induction of neuroprotective cytokines and inhibits hypoxia-inducing factor-1 (HIF-1alpha) [11], [14], [15]. Because the Mas receptor is found in high quantities within the hippocampus and perirhinal cortex as well as in vascular endothelial cells, PNA5 will be particularly effective in targeting memory impairments associated with hypoxia and inflammation-related neurodegenerative disease [16].

Our research team has previously shown in our preclinical model of VCID that 3 weeks of treatment with subcutaneous PNA5 reverses cognitive dysfunction [1], [3], [16] as measured by the Novel Object Recognition (NOR) and water maze tests, and is blocked by the selective MasR antagonist A779. Further, our preclinical studies have shown that 21-day treatment with PNA5 increases putative brain neuroprotective cytokines and inhibits circulating inflammatory cytokines and microglia activation, improves cerebral blood flow and blood-brain-barrier (BBB) integrity [3], [7].

To advance PNA5 to clinical trials to treat VCID in humans, we have completed studies required for submission of an Investigational New Drug (IND) to the FDA. The present study identifies the toxicity profile of PNA5 at 1, 5, and 40 mg/kg/day in rats and at 1, 5, and 20 mg/kg/day in dogs for 28 days.

## 2 Methods

These studies were performed in accordance with the U.S. Department of Health and Human Services, Food and Drug Administration, United States Code of Federal Regulations, Title 21, Part 58: Good Laboratory Practice for Nonclinical Laboratory Studies and as accepted by Regulatory Authorities throughout the European Union (OECD Principles of Good Laboratory Practice), Japan (MHLW), and other countries that are signatories to the OECD Mutual Acceptance of Data Agreement.

### 2.1 Chemicals and reagents

Manufacturing of GMP Glycosylated Angiotensin-(1−7) (PNA5) was performed by the PolyPeptide Group (San Diego, CA) via solid-phase synthesis, using methods previously described [1], [2], [17], The vehicle used for this study was 0.9 % sodium chloride (saline) for injection, USP.

#### 2.1.1 Stability

For the time periods covered by the parameters of this study, stability was demonstrated for all samples related to bulk test article, test article formulations, and bioanalysis. The analyzed dosing formulations were within the protocol-specified range of target concentrations for solutions and were stable at 0.1 mg/mL and 10 mg/mL following 24 h of room temperature storage and following 9 days of refrigerated storage.

#### 2.1.2 Dosing and dose formulation analyses

The analyzed dosing formulations contained 95.5–108.7 % of the test article, which was within the protocol-specified range of target concentrations for solutions (90–110 %), and were stable at 0.1 mg/mL and 10 mg/mL following 24 h of room temperature storage and following 9 days of refrigerated storage. The test article was not detected in the analyzed vehicle formulation that was administered to the control groups.

### 2.2 Animal study design

Animal studies and handling were conducted by Charles River Laboratories Ashland, LLC. All animal protocols (Rats: protocol number 00690526; Dogs: protocol number 00690527) and experiments used for these studies were approved by and adhered to Charles River Ashland Institutional Animal Care and Use Committee (IACUC). The care and use of animals was conducted in accordance with the guidelines of the USA National Research Council. Males and females were chosen to determine if there were sex-related differences in exposure and/or general toxicity. The total number of animals used in this study was considered to be the minimum required to properly characterize the effects of the test article. The planned dosing regimens in rats and dogs were designed to cover sub-therapeutic to supra-therapeutic levels to establish systemic and local safety margins.

#### 2.2.1 Sprague Dawley rats

Sprague Dawley rats (Crl:CD(SD)), approximately 8 weeks of age (176–302 g males, 130–217 g females) were supplied by Charles River Laboratories. Rats were housed (2–4 animals per cage) in a temperature, humidity, and light cycle-controlled facility. Rats were fed a chow diet (PMI Nutrition International, LLC LabDiet Certified CR Rodent Diet 5CR4); food and water were accessible to animals ad libitum.

Rats used in studies were inspected upon receipt for good health and were acclimated for 7 days at a minimum. Male and female rats were randomly selected for one of the four treatment groups upon receipt. Group 1 rats received vehicle control (0.9 % sodium chloride (saline) for injection, USP) subcutaneously for 28 days. Rats in groups 2–4 received PNA5 at 1, 5, or 40 mg/kg/day subcutaneously for 28 days (Table 1). Rats for all studies were dosed once daily for 28 consecutive days via subcutaneous bolus injection. The first day of dosing was based on day 1 body weights. Toxicokinetic study animals were dosed in the same fashion as for the main and recovery study animals.

**Table 1:** Sprague Dawley rats toxicology and toxicokinetic studies methodology- Table 1 illustrates the 28-day toxicology and toxicokinetic study design in Sprague Dawley rats. The dose was based on the most recent body weight measurement. The first day of dosing was based on day 1 body weights. Animals were dosed once a day subcutaneously for 28 days. Toxicokinetic study animals were dosed in the same fashion as for the main and recovery study animals. Animals in the main study were euthanized on day 29. For animals that were part of the recovery study, the first day of the recovery period was day 29 after the 28 days of treatment. Animals, a part of the recovery study, were euthanized on day 57.

**Group No.**	**Test Article**	**Dose Level**	**Dose Volume**	**Dose Concentration**	**Number of Animals**					
					**Toxicology** **Main Study**		**Toxicology Recovery Study**		**Toxicokinetic** **Study**	
		**(mg/kg/day)**	**(mL/kg)**	**(mg/mL)**	**Males**	**Females**	**Males**	**Females**	**Males**	**Females**
1	Vehicle Control	0	10	0	10	10	5	5	3	3
2	PNA−5	1		0.1	10	10	NA	NA	6	6
3	PNA−5	5		0.5	10	10	NA	NA	6	6
4	PNA−5	40		4	10	10	5	5	6	6

Rats are observed at least twice daily (morning and afternoon) beginning upon arrival through termination/release for mortality. For the toxicology main study, 10 rats per sex were randomly assigned to groups 1–4. 5 rats per sex were assigned to groups 1 (control) and group 4 (PNA5 at 40 mg/kg/day) for the toxicology recovery study. The number of animals used for the toxicokinetic groups was calculated to meet a sufficient number of animals to provide samples for toxicokinetic analysis while minimizing the number of blood collections per animal. For toxicokinetic studies, a total of 6 rats for each sex were assigned randomly to treatment groups 2–4 (those treated with PNA5). Three rats per sex were assigned to group 1 (vehicle control). Rats were sampled in satellite groups; rats' blood was taken at alternating time points, resulting in a total n = 3 per time point for six animals.

After 28 treatments, animals that were a part of the main and toxicokinetic study were scheduled for euthanasia on day 29, during which the necropsy measures were collected for the main study animals. Rats that were a part of the recovery animal study started the recovery period on day 29 and were scheduled for euthanasia on day 57, during which the same necropsy measures were collected. Animals were euthanized for humane reasons or at study termination via carbon dioxide inhalation followed by exsanguination via the vena cava for necropsy. Main Study and Recovery animals were fasted overnight prior to the scheduled necropsy. Following the final blood collection, animals in the toxicokinetic studies were euthanized by inhalation of carbon dioxide but were not exsanguinated for necropsy.

The following parameters and end points were evaluated in this study: mortality, clinical observations, body weights, body weight gains, food consumption, ophthalmology, clinical pathology parameters (hematology, coagulation, clinical chemistry, and urinalysis), toxicokinetic parameters, organ weights, and macroscopic and microscopic examinations.

#### 2.2.2 Beagle dogs

Beagles, approximately 7 months of age (7.6–9.5 kg males, 5.6–7.9 kg females) were supplied by Marshall BioResources. Animals were identified by tattoo. Dogs were group housed (up to 3 of the same sex in the same dosing groups per cage) in a temperature, humidity, and light cycle-controlled facility. Dogs were fed a chow diet (PMI Nutrition International, LLC LabDiet Certified Canine Diet 5007), approximately 300 g 1 h prior to dosing, with access to food for 3 h each day. Municipal tap water, treated by reverse osmosis and ultraviolet irradiation, was freely available.

Canines used in studies were inspected upon receipt for good health and had documentation of immunization for parvovirus, distemper, adenovirus type 2, parainfluenza, Bordetella, papilloma, and rabies. Animals were acclimated for 10 days at a minimum. Males and females were randomized separately to achieve similar group mean body weights. Final animal allocation was verified to ensure that littermates are homogeneously distributed across all groups. Four treatment groups were assigned; treatments were delivered as a subcutaneous bolus injection into the dorsothoracic region (Table 2). Group 1 dogs received vehicle control (vehicle control, 0.9 % sodium chloride for injection, pH 7) subcutaneously for 28 days. Dogs in groups 2–4 received PNA5 at 1, 5, and 20 mg/kg/day subcutaneously for 28 days. Dogs for all studies were dosed once daily for 28 consecutive days via subcutaneous bolus injection. Three dogs per sex were used in each treatment group in the main toxicology study. Two dogs per sex in group 1 (vehicle control) and group 4 (PNA5 20 mg/kg/day) were followed for the toxicology recovery study. The same animals for the main and recovery study were also used in the toxicokinetic studies, resulting in n of 3–5 animals per sex per group.

**Table 2:** Beagle dog toxicology and toxicokinetic studies methodology**-**Table 2 illustrates the 28-day toxicology study design in Beagle dogs. The dose volume was based on the most recent body weight measurement. The first day of dosing was based on day 1 body weights. Animals were dosed once a day subcutaneously for 28 days. Animals in the main study were euthanized on day 29. For animals that were part of the recovery study, the first day of the recovery period was day 29 after the 28 days of treatment. Animals, a part of the recovery study, were euthanized on day 43. The same dogs used in the toxicology main and recovery studies are the same as those used in the toxicokinetic studies.

**Group No.**	**Test Article**	**Dose Level**	**Dose Volume**	**Dose Concentration**	**Number of Animals**					
					**Toxicology** **Main Study**		**Toxicology Recovery Study**		**Toxicokinetic** **Study**	
		**(mg/kg/day)**	**(mL/kg)**	**(mg/mL)**	**Males**	**Females**	**Males**	**Females**	**Males**	**Females**
1	Vehicle Control	0	2	0	3	3	2	2	5	5
2	PNA−5	1	0.1	10	3	3	NA	NA	3	3
3	PNA−5	5	0.5	10	3	3	NA	NA	3	3
4	PNA−5	20	2	10	3	3	2	2	5	5

After 28 treatments, animals part of the main study were scheduled for euthanasia on day 29, during which the necropsy measures were collected. Dogs that were a part of the recovery animal study started the recovery period on day 29 and were scheduled for euthanasia on day 43, during which necropsy measures were collected. Dogs were euthanized for humane reasons or at study termination. Main Study and Recovery animals were fasted for at least 8 h (no more than 24 h) prior to the scheduled necropsy. Dogs were sedated with tiletamine/zolazepam powder (250 mg tiletamine/250 mg zolazepam) diluted with ketamine (100 mg/mL) and xylazine (100 mg/mL) administered intramuscularly prior to euthanasia via intravenous injection of sodium pentobarbital (130 mg/kg) and exsanguinated.

The following parameters and end points were evaluated in this study: mortality, clinical observations, body weights, body weight gains, food consumption, ophthalmology, electrocardiography, clinical pathology parameters (hematology, coagulation, clinical chemistry, and urinalysis), toxicokinetic parameters, organ weights, and macroscopic and microscopic examinations.

### 2.3 Parameters evaluated- clinical

#### 2.3.1 Daily observation

All animals were observed at least twice daily (morning and afternoon) beginning upon arrival through termination/release for mortality. Animals were observed daily. On days with dosing, observations were made 10–30 min postdosing. During the dosing, observations included but were not limited to the following: Changes in skin on the whole animal and at the injection site, fur, eyes, mucous membrane, and respiratory functions.

#### 2.3.2 Detailed physical examination

A detailed clinical observation was performed 1 week prior to randomization, on the date of randomization, and then weekly during the study period, with the last observation on the day of the scheduled necropsy.

#### 2.3.3 Injection site observations

The injection site was shaved and marked for identification and evaluation during the study. The injection site was collected at necropsy. Injection sites were observed for dermal findings, including erythema and edema.

#### 2.3.4 Individual body weights and food consumption

Rats were weighed within 4 days of receipt, on the day of randomization, daily during the study, and on the day of the scheduled necropsies. Dogs were weighed one week prior to randomization (± 2 days), on the day of randomization, on Day 1 (prior to dosing), weekly (± 2 days) during the study period, on the day prior to the scheduled necropsies, and on the day of the scheduled necropsy.

Rats' food consumption was measured quantitatively once weekly beginning on Day 1 and persisting throughout the study. Dogs' food consumption was measured daily beginning on Day 1 and persisting throughout the study.

#### 2.3.5 Ophthalmic examinations

Ophthalmic examinations were conducted by a board-certified veterinary ophthalmologist using an indirect ophthalmoscope and slit lamp biomicroscope. Prior to examination, animals will be treated with a mydriatic agent. Ophthalmic examinations were performed during the pretreatment period, near the end of the dosing period (Day 26 ± 2 days), near the end of the recovery period, if necessary, if findings are noted at the end of the dosing period

#### 2.3.6 Electrocardiograph and heart rates (Canines only)

ECGs were performed once during the pretreatment period, and near the end of the dosing period (Day 26 ± 2 days) for all animals in the main and recovery study. For animals in the recovery study, additional ECGs were performed near the end of the recovery period (Day 40 ± 2 days), and prior to recovery necropsy.

ECGs and heart rates were recorded on unfasted animals using the multi-lead ECG equipment (Standard 6 + 1 Lead ECG) with the DSI PONEMAH Physiology Platform (P3 Plus) version 5.0. ECG: V2, I, II, III, aVR, aVL, aVF lead data were collected. Recordings were taken continuously for at least 1 min. Quantitative ECG waveform analysis was performed using the DSI PONEMAH Physiology Platform (P3 Plus), version 5.0 (or higher) with ECG Template Analysis Option and ECG PRO to determine the heart rate, PR, QRS, RR, and QT intervals. Heart rate corrected QT (QTc) values were calculated with the Van de Water correction formula [18], [19]. Data were qualitatively evaluated by a veterinary cardiologist to detect rhythm or conduction disturbances or other abnormalities of the P-QRS-T waves.

### 2.4 Clinical pathology

Blood samples for clinical pathology were collected prior to treatment and on day 28. Blood samples were collected via venipuncture and used for the analysis of blood chemistry, hematology, coagulation, and serum chemistry parameters. Prior to collecting blood samples for clinical chemistry, animals were fasted for 8 h. Clinical chemistry parameters analyzed include: alanine aminotransferase, aspartate aminotransferase, alkaline phosphatase, gamma-glutamyl transferase, creatine kinase, total bilirubin, urea nitrogen, creatinine, calcium, phosphorus, total protein, albumin, globulin (calculated), albumin/globulin ratio, glucose, cholesterol, triglycerides, sodium, potassium, and chloride.

Urine was collected overnight. Rat urine was collected using metabolism cages, and dog urine was collected using cage pans. Urine was used to analyze urinalysis parameters, including color, appearance/clarity, specific gravity, pH, volume, protein, glucose, bilirubin, ketones, and blood.

#### 2.4.1 Anatomic pathology

Animals were subjected to a complete necropsy examination, which included evaluation of the carcass; all external surfaces and orifices; cranial cavity and external surfaces of the brain; and thoracic, abdominal, and pelvic cavities with their associated organs and tissues. Tissues and organs were collected during necropsy. Findings observed were considered incidental if the observations aligned with what is commonly seen in this animal strain and age, and/or were of similar incidence in control and treated animals, and, therefore, were considered unrelated to administration of the test article.

#### 2.4.2 Organ weights

Weighs of adrenals, brain, epididymides, heart, kidneys, liver, lungs, ovaries, pituitary, prostate, spleen, testes, thymus, thyroid with parathyroids, and uterus were taken at necropsy. Paired organs were weighed together.

#### 2.4.3 Microscopic evaluation

At necropsy, representative samples of tissues were collected and preserved in 10 % neutral buffered formalin. Tissues included for histopathological evaluation from dogs represent all major organ systems including but not limited to the cardiovascular (heart), nervous (brain, spinal cord, peripheral nerves, ganglia), lymphatic/immune (spleen, thymus, lymph nodes, bone marrow), respiratory (lungs, trachea), digestive (stomach, intestines, liver), endocrine (thyroid, adrenal), musculoskeletal (bone, joint, muscle), urogenital (kidneys, urinary tract), integumentary (skin, injection sites), reproductive organs (prostate, other female and male reproductive organs), and special sense organs (eye). In dogs, additional tissues, including glands (mammary glands, pituitary, parathyroid, and pancreas), were evaluated. Hematoxylin-eosin-stained paraffin sections were prepared for the tissues examined. Slides were evaluated histopathologically by light microscopy. Severity grades assigned to histopathologic changes in this study followed a 5-level severity grade scale of minimal, mild, moderate, marked, and severe.

### 2.5 Toxicokinetic analysis

#### 2.5.1 Intervals and timepoints

Toxicokinetic (TK) of PNA5 was obtained from plasma following a single and repeated subcutaneous injection administration of PNA5 once daily for 28 days. Blood samples were collected on days 1 and 28 at the following time points: pre-dose and at approximately 5, 15, 30, 60, and 120 min post-dose (equivalent to 0.083, 0.25, 0.5, 1, and 2 h).

For the rat study, a total of 6 rats for each sex were assigned randomly to treatment groups 2–4 (those treated with PNA5). Three rats per sex were assigned to group 1 (vehicle control). Rats were sampled in satellite groups; rats' blood was taken at alternating time points, resulting in a total n = 3 per time point for six animals. For the dog study, three animals per sex were assigned to group 2 and group 3, and five animals per sex were assigned to group 1 and group 4. Blood was collected from canines via venipuncture.

#### 2.5.2 Blood collection

Blood was collected in heparinized tubes containing a protease inhibitor cocktail consisting of 4 mM 0-phenanthroline, 1.2 mM pepstatin A, and 0.5 M EDTA. Blood samples were centrifuged for 15 min at 2500 x g at room temperature. Plasma was transferred into pre-cooled, pre-labeled collection tubes, frozen immediately in liquid nitrogen/dry ice, and stored at −80 °C until analysis.

#### 2.5.3 PNA5 analysis

This study was performed in a laboratory that follows Good Laboratory Practice (GLP) Regulations (U.S. Department of Health and Human Services, Food and Drug Administration (FDA) United States Code of Federal Regulations, Title 21, Part 58) [20]. PNA5 levels were analyzed using ultra-high performance liquid chromatography (UHPLC) with tandem mass spectrometric (MS/MS) detection. Precision and accuracy, reproducibility, selectivity, carryover, recovery, matrix effects, dilution integrity, hemolysis, stock stability, and matrix stability were assessed prior to initiation of the validation. Samples (5 µL) were injected onto the column and separated using gradient elution. Target compounds were quantified by summing the height of previously identified fragment peaks in the LC-MS [3] spectra. The serum concentrations were estimated using a calibration curve, accounting for the dilution factors and the probe recoveries for each experiment. Standards were matrix-matched to samples and underwent the same sample preparation steps.

#### 2.5.4 Toxicokinetic analysis

A noncompartmental analysis was used for parameter estimation. For PNA5 in plasma, the extravascular model was used for parameter estimation. For the rat study, all parameters were generated from PNA5 individual concentrations in plasma from days 1 and 28. Parameters were estimated using nominal dose levels and nominal sampling times relative to the dose administration. Plasma concentration values obtained at the pre-dose time point were used as the concentration at time 0 on days 1 and 28. For the dog study, all parameters were generated from PNA5 individual concentrations in plasma from days 1 and 28. Parameters were estimated using nominal dose levels and nominal sampling times relative to dose administration. Plasma concentration values obtained at the pre-dose time point were used as the concentration at time 0 on days 1 and 28. For Beagle dogs, concentration values reported as below the limit of quantitation (< 5 ng/mL) were assigned a value of 0.

The toxicokinetic parameters evaluated included the area under the curve of the plasma PNA5 concentration against time from time 0 (pre-dose) to 2 h (post-dose) (AUC_0–2hr_), the maximum plasma concentration (C_max_), time to maximum plasma concentration (T_max_), elimination rate (Kel), and the half-life. The area under the concentration vs. time curve was calculated using the linear trapezoidal method with linear interpolation for all profiles with at least three consecutive quantifiable concentrations. Kel was calculated from the slope using exponential regression of concentration versus time (Excel); exponential interpolations for all profiles include at least three concentrations plotted against time. Time 0 values and all concentrations listed as zero were excluded from Kel calculations. The half-life was calculated from Kel via the following equation: half-life Ln(2)/Kel.

### 2.6 Safety data parameters

Safety parameters for PNA5-related pulmonary, neurobehavioral, and cardiovascular effects were also assessed.

Respiratory rate, tidal volume, and calculated minute volume were measured in male Sprague Dawley rats to measure pulmonary safety parameters after a single subcutaneous dose of 0, 1, 5, or 40 mg/kg PNA5 (n = 8 males per group).

To evaluate the effects of PNA5 when administered subcutaneously on the central nervous system’s autonomic, neuromuscular, sensorimotor, and behavioral domains, neurobehavioral evaluations were performed in male Sprague Dawley rats treated with PNA5 at 0, 1, 5, or 40 mg/kg (n = 8 males per group). The neurobehavioral evaluations consisted of assessments of the central nervous system for activity, excitability, autonomic function, neuromuscular function, sensorimotor responses, and physiological function.

PNA5 -related cardiovascular effects were evaluated in male Beagle dogs at dose levels of 1, 5, or 20 mg/kg. To evaluate the effects of PNA5 when administered subcutaneously on the cardiovascular system the following parameters were measured including clinical observations, heart rate, arterial blood pressure (systolic, diastolic, and mean), pulse pressure, body temperature, and ECG waveforms (from which ECG waveform morphologies and ECG intervals PR, QRS, QT, and heart rate corrected QT [QTc] were derived).

### 2.7 Statistics

#### 2.7.1 Clinical and pathology analysis

Comparisons of body weight gains and food consumption were calculated between data from days 1–28 for all studies and 28–57 days for the recovery study in rats and 28–43 days for the recovery study in dogs. Organ weight relative to body weight was calculated against the terminal body weight. Organ weight was also compared to brain weight. Levene’s test will be used to assess the homogeneity of group variances.

The groups will be compared using an overall one-way ANOVA F-test if Levene’s test is not significant or the Kruskal-Wallis test if it is significant. If the overall F-test or Kruskal-Wallis test is found to be significant, then pairwise comparisons will be conducted using Dunnett’s or Dunn’s test, respectively. Statistics were performed using SAS. P values ≤ 0.05 are considered significant.

#### 2.7.2 Toxicokinetic statistical analysis

Descriptive statistics (sample size [n], arithmetic mean, median (min–max), standard deviation [std], and coefficient of variation expressed as a percent [%CV]) and ratios (accumulation, sex, etc.) for appropriate grouping and sorting variables were generated. The bioanalytical data, TK parameters, ratios, and descriptive statistics were reported to 2 significant figures, as well as the n, which was reported with no decimal.

Values were tested for normality via Shapiro-Wilk test. Sex related concentration differences for each timepoint (t = 0, 0.083, 0.25, 0.5, 1, and 2 h) and dose were compared to the corresponding timepoint and dose concentration of the opposing sex within the same testing date via unpaired *t*-test if values met normality, or the Mann-Whitney test for values that did not meet normality. To determine differences in concentration over treatment time (day 1 to day 28), values were compared using a paired *t*-test if the data sets were normal, or a Wilcoxon test for non-normal data sets. Comparison of toxicokinetic parameters across doses was tested for significance via one-way ANOVA F-test if values are normal or the Kruskal-Wallis test for non-normal data sets. P values ≤ 0.05 are considered significant.

## 3 Results

### 3.1 Toxicology studies

For both Sprague-Dawley rats and Beagle dogs in the main and recovery studies, once daily subcutaneous bolus injection for 28 days of PNA5 (with 0.9 % sodium chloride, pH 7, for injection as the vehicle) was well tolerated at all doses with no effects on mortality or adverse findings. There were no test article-related effects on survival, clinical observations, dermal observation, body weight, food consumption, clinical pathology, macroscopic observation, organ weights, or microscopic evaluations.

#### 3.1.1 Sprague Dawley rat

All animals survived until the scheduled euthanasia at all doses, 1, 5, and 40 mg/kg/day. There were no test article-related clinical observations or effects on body weight, food consumption, electrocardiography, hematology, coagulation, clinical chemistry, urinalysis, or organ weights.

##### 3.1.1.1 Clinical observations

There were no test article-related clinical observations for all dose levels (1, 5, 40 mg/kg/day subq for 28 days) in both the main and recovery study. All clinical observations in the test article-treated groups were noted with similar incidence in the control group, were limited to single animals, were not noted in a dose-related manner, and/or were common findings for laboratory rats of this age and strain. No ophthalmic lesions indicative of toxicity were observed in any of the test article-treated groups. Body weights and body weight gains were unaffected by test article administration. Food consumption was unaffected by test article administration. There were no notable clinical observation differences when the control and test article-treated groups were compared at all dose levels.

##### 3.1.1.2 Clinical pathology (Day 29, main study)

No clinical pathology was observed for the main study for all dose levels. Hematology, coagulation, clinical chemistry, and urinalysis parameters were unaffected by test article administration.

There were no test article-related macroscopic findings. However, dark red subcutaneous foci were noted at the subcutaneous injection sites in both the control and treated groups of female animals. Injection site observations lacked a dose response. These injection site observations were at all dose levels; therefore, these observations at the injection site were considered an effect of subcutaneous injection rather than a direct toxic effect of the test article.

There were no test article-related organ weight changes. Organ weight values were not significantly different from their respective controls.

There were no test article-related microscopic findings. Of note, while there was an increase in incidence and severity of mixed cell infiltration at the subcutaneous dorsoscapular injection site in 40 mg/kg/day group females, the findings were all considered within the expected response to the procedure of injection [21]. Other microscopic findings observed were considered incidental, of the nature commonly observed in this strain and age of rats, and/or were of similar incidence and severity in control and treated animals and, therefore, were considered unrelated to administration of the test article.

##### 3.1.1.3 Clinical pathology (Day 43, recovery study)

There were no test article-related macroscopic findings. No injection site macroscopic observations were noted.

There were no test article-related organ weight changes. There were no significant differences in any organ weight when comparing the mean organ weight of treated animals to that of controls at euthanasia. However, when normalized to body weight or brain weight, the 40 mg/kg/day group males' prostate/seminal vesicle normalized weights were significantly higher than the control animals' normalized prostate/seminal vesicle weights. These results are considered incidental, unrelated to the administration of the test article, as the absolute mean prostate/seminal vesicle weights were not significantly different from concurrent controls. Further, there were no statistically significantly different mean prostate/seminal vesicle weights at the terminal euthanasia, and the mean weights remained within the mean ±2 standard deviation values in the Charles River Ashland historical control database [22].

No test article-related microscopic findings were noted in the gross observation evaluated.

#### 3.1.2 Beagle dogs

All animals survived until the scheduled euthanasia at all doses, 1, 5, and 20 mg/kg/day. There were no test article-related clinical observations or effects on body weight, food consumption, electrocardiography, hematology, coagulation, clinical chemistry, urinalysis, or organ weights. Based on these results, the no-observed-adverse-effect-level (NOAEL) was considered to be 20 mg/kg/day.

##### 3.1.2.1 Clinical observation

There were no test article-related clinical observations. All clinical observations in the test article-treated groups were noted with similar incidence in the control group, were limited to single animals, were not noted in a dose-related manner, and/or were common findings for laboratory dogs of this age and breed.

Body weights and body weight gains were unaffected by test article administration. Food consumption was unaffected by test article administration. No ophthalmic lesions indicative of toxicity were observed in any of the test article-treated groups. All findings observed were typical in prevalence and appearance for laboratory dogs of this age and breed.

##### 3.1.2.2 Clinical pathology (Day 29, main study)

Hematology, coagulation, clinical chemistry, and urinalysis parameters were unaffected by test article administration. No test article-related macroscopic findings were noted. Any macroscopic findings observed were considered incidental, of the nature commonly observed in this strain and age of rats, and/or were of similar incidence in control and treated animals, and, therefore, were considered unrelated to administration of the test article.

No test article-related macroscopic findings were noted globally. As a result of the injection, at the injection site, dark red subcutaneous foci were noted in 5 of 96 subcutaneous injection sites in the treated group animals but lacked a dose response. These injection site observations were seen at low incidences (1 per injection site per group, when present) at all dose levels; therefore, these observations at the injection site were considered an effect of subcutaneous injection rather than a direct toxic effect of the test article. All remaining macroscopic findings observed were considered incidental, of the nature commonly observed in this age of dogs, and/or were of similar incidence in control and treated animals and, therefore, were considered unrelated to administration of the test article.

No test article-related microscopic findings were noted globally. As a result of the injection, not the treatment itself, minimal to moderate subcutaneous mixed cell infiltrate or inflammation characterized by low to moderate numbers of macrophages, neutrophils, lymphocytes, and/or plasma cells, or minimal to mild mononuclear cell infiltration or inflammation were present at one or more subcutaneous injection sites. These observations were present in both the PNA5-treated animals and the control groups. Mixed cell and mononuclear cell infiltration and inflammation were considered a continuum of inflammatory changes, and because they occurred in both control and treated groups, they were determined to be an effect of the injection process rather than an effect of test article administration. The remaining microscopic findings observed were considered incidental, of the nature commonly observed in this age of dogs, and/or were of similar incidence and severity in control and treated animals and, therefore, were considered unrelated to administration of the test article.

##### 3.1.2.3 Clinical pathology (Day 43, recovery study)

No test article-related macroscopic findings were noted. The macroscopic findings observed were considered incidental, of the nature commonly observed in this age of dogs, and/or were of similar incidence in control and treated animals and, therefore, were considered unrelated to administration of the test article. There were no test article-related organ weight changes. No test article-related microscopic findings were noted. The microscopic findings observed were considered incidental, of the nature commonly observed in this age of dogs, and/or were of similar incidence in control and treated animals and, therefore, were considered unrelated to administration of the test article.

### 3.2 Toxicokinetic studies

#### 3.2.1 Sprague Dawley rat

**Fig. 1:**
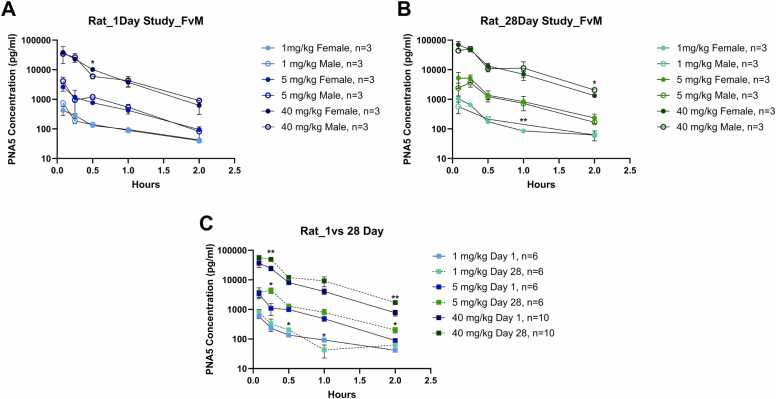
PNA5 concentration curve in Sprague Dawley rat plasma. For all graphs, there are three injection concentrations: 1 mg/kg, 5 mg/kg, and 40 mg/kg; colors for the increasing concentrations are represented by an increase in color saturation. Injection concentrations on day 1 are represented by blue symbols and are designated the following colors: 1 mg/kg day 1 –light blue, 5 mg/kg day 1- royal blue, 40 mg/kg day 1- black blue. Injection concentrations on day 28 are represented by green symbols and are designated the following colors: 1 mg/kg day 1 –light green, 5 mg/kg day 1- leaf green, 40 mg/kg day 1- dark green. Plasma PNA5 concentration (pg/mL) in rats comparing Female vs Male for **A)** day 1 and **B)** day 28 are plotted against time (hours). Females are represented by filled circles, and males are represented by hollow circles. Combined sexes are represented by filled squares. Plasma PNA5 concentrations in rats (female and male values combined), comparing **C)** day 1 vs 28, are plotted against time. Significance between comparative groups at each time point was tested via a student's *t*-test. Significance is indicated via *. * p ≤ 0.05 **p ≤ 0.005. The Y axis is log-transformed.

Plasma concentrations of PNA5 were determined using a high-performance liquid chromatography assay. Rats in group 1 (control) plasma PNA5 concentration levels were below detection. For animals dosed with PNA5 (Groups 2–4), pre-dose plasma PNA5 concentrations were below detection limits for both sexes on days 1 and 28. Post-dose PNA5 plasma concentrations on day 1 and day 28 were quantifiable up to 2 h for animals dosed 1, 5, or 40 mg/kg/day (Groups 2–4, respectively). Average T_max_ values for PNA5 in plasma concentrations were observed by 0.11, 0.083, 0.14 h post-dose at 1, 5, and 40 mg/kg/day on day 1 and 0.17, 0.19, 0.17 h post-dose at 1, 5, 40 mg/kg/day on day 28 (individual T_max_ values for PNA5 ranged from 0.083 to 0.25 h post-dose on day 1 and day 28). PNA5 plasma concentrations are summarized in Fig. 1.

Systemic exposure to PNA5 appeared to be independent of sex (Figs. 1**A,** 1**B**). While there were some differences between sexes at different time points, there was no consistent difference in overall concentration time profiles across treatments or dosing days. AUC_0–2hr_, C_max_, and Kel show no consistent sex differences. Plasma PNA5 AUC_0–2hr_ values on day 1 for females ranged from 253.40 ± 38.86, 3–16469.00 ± 5417.59 hr*pg/mL and males AUC values ranged from 272.17 ± 26.70–15706.33 ± 4854.81 hr*pg/mL, and for day 28, females AUC values ranged from 498.59 ± 64.2, 3–29563.58 ± 5124.59 hr*pg/mL and males ranged 428.56 ± 76.84–29707.30 ± 12,919.68 hr*pg/mL. The F:M AUC_0–2hr_ on day 1 ranges from 0.93 to 1.16, 0.66–1.58, and 1.16–1.32 for the corresponding doses of 1, 5, and 40 mg/kg/day. F:M C_max_ ratios on day 1 ranged from 0.58 to 1.16 and 1.24 – 1.37 on day 28. There were no consistent differences in individual concentration-time profiles, C_max_, and AUC values; therefore, the following discussion is based on data for males and females combined.

Following once daily administration of PNA5 to rats, C_max_ and AUC_0–2hr_ values of PNA5 increased with increasing dosages on days 1 and 28. A 40-fold increase in PNA5 dose resulted in an approximate 69.09-fold increase in PNA5 C_max_ values on day 1, and a 64.56-fold increase on day 28. Similarly, a 40-fold increase in PNA5 dose resulted in an approximate 61.22-fold increase in PNA5 AUC_0–2hr_ values on day 1, and a 63.93-fold increase on day 28. TK profiles appeared to be similar across treatment groups. Following once daily SC administration of PNA5 to rats, C_max_ and AUC_0–2hr_ values of PNA5 increased with increasing dose on days 1 and 28. C_max_/Dose (Table 3) range from 590.62 ± 250.36–1020.21 ± 513.76 for day 1, and 968.15 ± 280.22–1562.51 ± 612.034 for day 28. AUC_0–2hr_/Dose values range from 262.78 ± 31.54–402.19 ± 115.49 for day 1, and 463.57 ± 74.06–740.89 ± 219.77 for day 28.

**Table 3:** Sprague Dawley rats toxicokinetic summary**-** Toxicokinetic values for rats dosed with PNA5 are presented for all three concentrations, 1, 5, 40 mg/kg/day for days 1 and 28. Animals were treated once daily subcutaneously for a total of 28 days. No sex differences were accounted for, so means and standard deviations represent both sexes. Values are presented as the average mean ± standard deviation (SD), and the number of animals within each group. T_max_ is presented as the average, followed by the range of the lowest to the highest time point for T_max_. Significance between day 1 and day 28 toxicokinetic parameters was tested using a paired *t*-test if test values were normally distributed or a Wilcoxon test when not normally distributed (represented by #). * p ≤ 0.05 **p ≤ 0.005.

**Toxicokinetic-Rat**			
	**Day 1** **(Mean±SD, N = )**	**Day 28** **(Mean±SD, N = )**	**p-value**
**1 mg/kg/Day Dose**			
**C**_**max**_**(pg/mL)**	590.62 ± 250.36, 6	968.15 ± 280.22, 6	0.08
**C**_**max**_**/Dose (pg/mL/(mg/kg/day))**	590.62 ± 250.36, 6	968.15 ± 280.22, 6	0.08
**T**_**max**_**(hr)**	0.11(0.083–0.25)	0.17(0.083–0.25)	0.63#
**Half-life (hr)**	0.60 ± 0.10, 6	0.51 ± 0.15, 6	0.31#
**Kel (hr–1)**	1.18 ± 0.18, 6	1.45 ± 1.36, 6	0.18
**AUC**_**0–2**_(hr*pg/mL)	262.78 ± 31.54, 6	463.57 ± 74.06, 6	***0.0006
**AUC**_**0–2**_/Dose (hr*pg/mL/(mg/kg/day))	262.78 ± 31.54, 6	463.57 ± 74.06, 6	***0.0006
**5 mg/kg/Day Dose**			
**C**_**max**_**(pg/mL)**	3371.50 ± 1985.69, 6	5353.58 ± 2767.46, 6	0.25
**C**_**max**_**/Dose (pg/mL/(mg/kg/day))**	674.30 ± 397.14, 6	1070.72 ± 553.49, 6	0.25
**T**_**max**_**(hr)**	0.083	0.19(0.083–0.25)	*0.03
**Half-life (hr)**	0.42 ± 0.13, 6	0.37 ± 0.13, 6	0.57
**Kel (hr–1)**	1.79 ± 0.67, 6	2.08 ± 0.86, 6	0.69#
**AUC**_**0–2**_(hr*pg/mL)	1547.52 ± 788.68, 6	2863.37 ± 1720.52, 6	0.12
**AUC**_**0–2**_/Dose (hr*pg/mL/(mg/kg/day))	309.50 ± 157.74, 6	572.67 ± 344.10, 6	0.12
**40 mg/kg/Day Dose**			
**C**_**max**_**(pg/mL)**	40,808.50 ± 20,550.45, 6	62,500.20 ± 24,481.35, 6	*0.05
**C**_**max**_**/Dose (pg/mL/(mg/kg/day))**	1020.21 ± 513.76, 6	1562.51 ± 612.034, 6	*0.05
**T**_**max**_**(hr)**	0.14(0.083–0.25)	0.17(0.083–0.25)	0.36
**Half-life (hr)**	0.33 ± 0.08, 6	0.39 ± 0.06, 6	0.21
**Kel (hr–1)**	2.24 ± 0.83, 6	1.82 ± 0.28, 6	0.29
**AUC**_**0–2**_(hr*pg/mL)	16,087.63 ± 4619.77, 6	29,635.44 ± 8790.79, 6	**0.002
**AUC**_**0–2**_/Dose (hr*pg/mL/(mg/kg/day))	402.19 ± 115.49, 6	740.89 ± 219.77, 6	**0.002

Systemic exposure (AUC_0–2hr_ values) to PNA-5 in rats did appear to increase following repeated administration of PNA5 in both Females and Males (Fig. 1**C**- female and male combined). AUC_0–2hr_ averaged values were significantly higher for doses 1 and 40 mg/kg/day when comparing day 1 to day 28 (Table 3). The AUC_0–2hr_ averaged accumulation ratios were 1.76, 2.50, and 1.84 at 1, 5, and 40 mg/kg/day, respectively, from day 1 to day 28.

#### 3.2.2 Beagle dogs

**Fig. 2:**
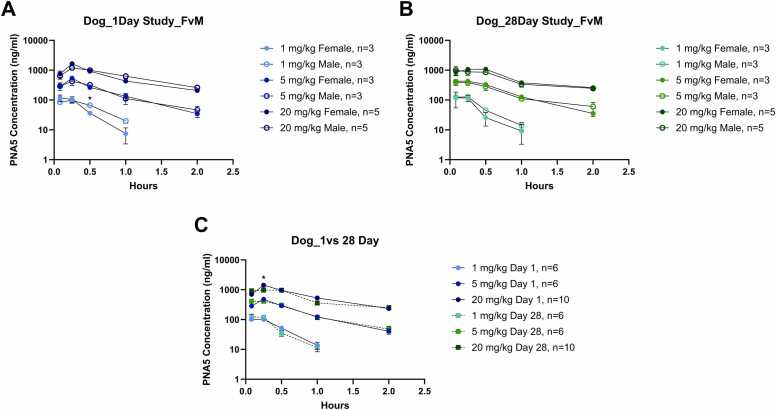
PNA5 concentration curve in Beagle dog plasma. For all graphs, there are three injection concentrations: 1 mg/kg, 5 mg/kg, and 20 mg/kg; colors for the increasing concentrations are represented by an increase in color saturation. Injection concentrations on day 1 are represented by blue symbols and are designated the following colors: 1 mg/kg day 1 –light blue, 5 mg/kg day 1- royal blue, 20 mg/kg day 1- black blue. Injection concentrations on day 28 are represented by green symbols and are designated the following colors: 1 mg/kg day 1 –light green, 5 mg/kg day 1- leaf green, 20 mg/kg day 1- dark green. Plasma PNA5 concentration (ng/mL) in dogs comparing female vs male for **A)** day 1 and **B)** day 28 are plotted against time (hours). Females are represented by filled circles, and males are represented by hollow circles. Combined sexes are represented by filled squares. Plasma PNA5concentrations in dogs (female and male values combined), comparing **C)** day 1 vs 28, are plotted against time. Significance between comparative groups at each time point was tested via a student's *t*-test. Significance is indicated via *. * p ≤ 0.05. The Y axis is log-transformed.

Male and female dogs were administered PNA5 daily by subcutaneous injection at dose levels of 1, 5, and 20 mg/kg/day for up to 28 days. The toxicokinetics of PNA5 were evaluated in plasma samples collected on days 1 and 28. PNA5 was quantifiable in dogs up to 1 h post-dose at 1 mg/kg/day and up to 2 h post-dose at 5 and 20 mg/kg/day on Days 1 and 28. The median T_max_ values for PNA5 in plasma concentrations were observed by 0.167 h post-dose at 1 mg/kg/day and 0.25 h post-dose at 5 and 20 mg/kg/day on Day 1 and 0.167 h post-dose at 1 and 5 mg/kg/day and 0.375 h post-dose at 20 mg/kg/day on Day 28 (individual T_max_ values for PNA-5 ranged from 0.083 to 0.5 h post-dose on Day 1 and Day 28). PNA5 plasma concentrations are summarized in Fig. 2.

Systemic exposure to PNA5 appeared to be independent of sex (Figs. 2**A,** 2**B**). There were no consistent differences in individual concentration-time profiles, C_max_ and AUC values (F:M C_max_ ratios ranged from 0.96 to 1.33 and F:M AUC_0–2hr_ ratios ranged from 0.79 to 1.14), therefore, the following results are based on data for males and females combined.

Following daily subcutaneous administration of PNA5 to dogs, C_max_ and AUC_0–2hr_ values of PNA5 increased with increasing dose in a less than dose-proportional manner for C_max_ and an approximately dose-proportional manner for AUC_0–2hr_ on days 1 and 28. A 20-fold increase in PNA5 dose resulted in an approximate 13.06-fold increase in PNA5 C_max_ values on day 1, and a 7.70-fold increase on day 28, with the C_max_/Dose decreasing with increased dosages (Table 4). A 20-fold increase in PNA5 dose resulted in an approximate 20.06-fold increase in PNA5 AUC_0–2hr_ values on day 1, and a 17.19-fold increase on day 28.

**Table 4:** Beagle dogs toxicokinetic summary**-** Toxicokinetic values for dogs dosed with PNA5 are presented for all three concentrations, 1, 5, 20 mg/kg/day for days 1 and 28. Animals were treated once daily subcutaneously for a total of 28 days. No sex differences were accounted for, so means and standard deviations represent both sexes. Values are presented as the average ± standard deviation (SD), and the number of animals within each group. T_max_ is presented as the average, followed by the range of the lowest to the highest time point for T_max_. Significance between day 1 and day 28 toxicokinetic parameters was tested using a paired *t*-test.

**Toxicokinetic -Dog**			
	**Day 1** **(Mean±SD, N = )**	**Day 28** **(Mean±SD, N = )**	**p-value**
**1 mg/kg/Day Dose**			
**C**_**max**_**(ng/mL)**	109.93 ± 37.34, 6	145.63 ± 59.63, 6	0.12
**C**_**max**_**/Dose (ng/mL/(mg/kg/day))**	109.93 ± 37.34, 6	145.63 ± 59.63, 6	0.12
**T**_**max**_**(hr)**	0.167(0.083–0.25)	0.167(0.083–0.25)	> 0.9999
**Half-life (hr)**	0.29 ± 0.10, 6	0.26 ± 0.08, 6	0.59
**Kel (hr**^**–1**^**)**	2.63 ± 0.97, 6	2.87 ± 0.86, 6	0.60
**AUC**_**0–2**_(hr*ng/mL)	63.47 ± 14.80, 6	62.61 ± 19.05, 6	0.89
**AUC**_**0–2**_/Dose (hr*ng/mL/(mg/kg/day))	63.47 ± 14.80, 6	62.61 ± 19.05, 6	0.89
**5 mg/kg/Day Dose**			
**C**_**max**_**(ng/mL)**	496.50 ± 115.46, 6	443.50 ± 101.46, 6	0.35
**C**_**max**_**/Dose (ng/mL/(mg/kg/day))**	99.30 ± 23.09, 6	88.70 ± 20.29, 6	0.35
**T**_**max**_**(hr)**	0.25(0.25–0.5)	0.167(0.083–0.5)	0.40
**Half-life (hr)**	0.50 ± 0.13, 6	0.58 ± 0.21, 6	0.10
**Kel (hr**^**–1**^**)**	1.44 ± 0.33, 6	1.32 ± 0.43, 6	0.09
**AUC**_**0–2**_(hr*ng/mL)	358.23 ± 95.10, 6	365.12 ± 42.57, 6	0.81
**AUC**_**0–2**_/Dose (hr*ng/mL/ (mg/kg/day))	71.68 ± 19.02, 6	73.02 ± 8.51, 6	0.81
**20 mg/kg/Day Dose**			
**C**_**max**_**(ng/mL)**	1435.30 ± 452.69, 10	1121.30 ± 530.40, 10	0.11
**C**_**max**_**/Dose (ng/mL/(mg/kg/day))**	71.77 ± 22.63, 10	56.07 ± 26.52, 10	0.11
**T**_**max**_**(hr)**	0.25(0.25–0.5)	0.375(0.083–0.5)	0.34
**Half-life (hr)**	0.69 ± 0.10, 10	0.81 ± 0.21, 9	0.15
**Kel (hr**^**–1**^**)**	1.02 ± 0.17, 10	0.86 ± 0.35, 10	0.24
**AUC**_**0–2**_(hr*ng/mL)	1273.15 ± 269.76, 10	1076.09 ± 308.76, 10	0.11
**AUC**_**0–2**_/Dose (hr*ng/mL/(mg/kg/day))	63.66 ± 13.49, 10	53.80 ± 15.44, 10	0.11

Systemic exposure (C_max_ and AUC_0–2hr_ values) to PNA5 in dogs was similar on days 1 and 28 following repeated administration of PNA5 (Fig. 2**C**- female and male combined). Systemic exposure (AUC_0–2hr_ values) to PNA5 in dogs did not appear to change following repeated administration of PNA5. AUC_0–2hr_ accumulation ratios were 0.99, 1.02, and 0.85 at 1, 5, and 20 mg/kg/day, respectively.

Based on these results, the no-observed-adverse-effect-level (NOAEL) in dogs was considered to be 20 mg/kg/day. This dose corresponded to mean AUC_0–2hr_ values of 1147.86 and 1004.32 hr*ng/mL and mean C_max_ values of 1142.00 and 1100.60 ng/mL for females and males, respectively, on Day 28.

### 3.3 Safety data summary

In Safety Data Studies, single administration of PNA5 at the administered doses had no effects on pulmonary, neurobehavioral, and cardiovascular measured parameters.

Single subcutaneous injection of PNA5 at 0, 1, 5, or 40 mg/kg in male Sprague Dawley rats resulted in no PNA5-related respiratory or neurobehavioral parameters. PNA5 had no effect on the respiratory rate, tidal volume, and calculated minute volume; nor were there observed PNA5-related effects on assessments of the central nervous system for activity, excitability, autonomic function, neuromuscular function, sensorimotor responses, and physiological function.

Single subcutaneous injection of PNA5 at 0, 1, 5, or 20 mg/kg in male Beagle dogs resulted in no PNA5-related cardiovascular parameters. PNA5 had no effect on clinical observations, heart rate, arterial blood pressure (systolic, diastolic, and mean), pulse pressure, body temperature, and ECG waveforms.

## 4 Discussion

This 28-day repeat-dose toxicology study demonstrates that subcutaneous administration of PNA5, a glycosylated Angiotensin-(1−7) analog with Mas receptor agonism, was well tolerated in both Sprague Dawley rats and Beagle dogs. Across all dose levels tested, there were no treatment-related effects on mortality, clinical signs, or systemic or local toxicity in either species. These findings complement earlier pharmacodynamic data in mouse models of vascular cognitive impairment and dementia (VCID), in which PNA5 significantly reduced neuroinflammation, reactive oxygen species, and cerebrovascular injury, while restoring cerebral blood flow and cognitive function [1], [7].

Toxicokinetic (TK) results revealed that PNA5 systemic exposure increased with dosing in both rats and dogs, though the relationship differed by species. In both species, PNA5 pharmacokinetics were independent of sex, and exposure-time profiles were generally consistent between individuals.

In rats, C_max_, and AUC_0–2hr_ increased approximate to dose proportionally on both day 1 and day 28. Notably, systemic exposure in rats was higher on day 28 compared to day 1, suggesting limited accumulation or possible saturation of clearance mechanisms at higher exposures.

Results from the dog study found that C_max_ increased less than dose proportionally, and AUC_0–2hr_ increased approximately dose proportionally on day 1 and day 28, with no indication of accumulation.

These species differences in systemic exposure and accumulation may be partially explained by known interspecies variation in the metabolism of Ang-(1−7) peptides and the activity of relevant proteolytic enzymes, including ACE1 and ACE2 [23], [24], [25]. Ang-(1−7) is generated via ACE2-mediated conversion of Angiotensin II or via neprilysin activity on Angiotensin I and is rapidly degraded by ACE1 and other aminopeptidases [24], [26], [27]. Rats exhibit high pulmonary and systemic ACE1 activity, which can lead to rapid degradation of Ang-(1−7) and its analogs [23], [24]. Furthermore, rat plasma is rich in peptidases such as aminopeptidase A and prolyl endopeptidase that also contribute to shorter half-lives for small peptides.

In contrast, dogs have comparatively lower systemic ACE1 activity and higher ACE2 expression in organs such as the kidney and vasculature [23], [28]. This enzymatic profile may favor preservation of circulating Ang-(1−7) analogs and help explain the more stable and consistent exposure patterns observed in Beagle dogs. The glycosylation of PNA5 may further enhance metabolic stability by reducing protease susceptibility, though this protective effect likely varies by species depending on local enzymatic expression and tissue distribution.

However, these species differences in ACE activity alone do not account for why accumulation was seen in the rats but not in the dogs. Similar differences in Ang-(1−7) accumulation were also observed by Mordwinkin et al [29]. A second hypothesis for why species differences in accumulation were observed is that chronic administration over 28 days may result in local or systemic enzyme saturation, particularly in species with initially high peptidase activity [30], [31]. In rats, this could reduce metabolic clearance efficiency over time, leading to the accumulation of PNA5. Alternatively, species differences in tissue distribution or depot formation at the subcutaneous injection site—more common in rats due to higher subcutaneous vascularization and fat content—may result in slow release and delayed systemic clearance [32]. In contrast, dogs may maintain consistent clearance rates without enzyme saturation or depot effects, accounting for the lack of observed accumulation.

Understanding these interspecies enzymatic differences is crucial for translational development. Human ACE2 expression is more restricted than in rodents and is primarily localized to the lung, heart, kidney, and gastrointestinal tract. Moreover, human ACE1 and ACE2 activities differ substantially from those of rodents and more closely resemble the enzymatic milieu observed in dogs [25]. These factors reinforce the value of Beagle dogs as a more predictive nonclinical model for assessing the pharmacokinetics and metabolic stability of Ang-(1−7) analogs such as PNA5.

In summary, these data confirm that PNA5 is well tolerated in both rats and dogs at all tested doses. The observed species-specific differences in systemic exposure and accumulation reflect underlying variation in the metabolism of Ang-(1−7) and expression of its regulating enzymes. These findings support further development of PNA5 and provide confidence in the selection of the dog model for translational toxicology and pharmacokinetic extrapolation to humans.

## Authors contributions

M.H. conceived the study and obtained research fundings. C.H-K., J.P.K., and M.H. made contributions to all of the following: (1) analysis, or interpretation of data; and (2) approved the final version to be submitted. C.H-K., and M.H. also made contributions to the manuscript by drafting the article and/or revising the manuscript for important intellectual content.

## CRediT authorship contribution statement

**Meredith Hay:** Writing – review & editing, Writing – original draft, Validation, Supervision, Project administration, Methodology, Investigation, Funding acquisition, Formal analysis, Conceptualization. **Christina Hoyer-Kimura:** Writing – review & editing, Writing – original draft, Visualization, Validation, Formal analysis. **John P. Konhilas:** Writing – review & editing, Investigation, Formal analysis.

## Ethical approval

All procedures, handling, and treatments were approved by the institutional Animal Care and Use Committee at the University of Arizona in agreement with the National Institute of Health Guidelines for the care and Use of Laboratory Animals.

## Funding

This study was funded by 10.13039/100000049NIA under Grant U01AG066623; and Grant U01AG082617.

## Disclosure statement

Competing interests: COI, Dr. Meredith Hay is the founder of ProNeurogen, Inc that holds exclusive rights to the clinical development of the Ang-(1−7)/MasR agonists described herein.

## Declaration of Competing Interest

The authors declare the following financial interests/personal relationships which may be considered as potential competing interests: Meredith Hay PhD reports financial support was provided by National Institute on Aging. Meredith Hay PhD has patent licensed to ProNeurogen,Inc. Competing interests: COI, Dr. Meredith Hay is the founder of ProNeurogen, Inc that holds exclusive rights to the clinical development of the Ang-(1−7)/MasR agonists described herein. If there are other authors, they declare that they have no known competing financial interests or personal relationships that could have appeared to influence the work reported in this paper.

## Data Availability

Data will be made available on request.
